# Effective Doping of Single-Walled Carbon Nanotubes with Polyethyleneimine

**DOI:** 10.3390/ma14010065

**Published:** 2020-12-25

**Authors:** Monika Rdest, Dawid Janas

**Affiliations:** 1 Department of Materials Science and Metallurgy, University of Cambridge, Cambridge CB3 0FS, UK; Monika.Rdest@gmail.com; 2 Department of Organic Chemistry, Bioorganic Chemistry and Biotechnology, Silesian University of Technology, 44-100 Gliwice, Poland

**Keywords:** carbon nanotubes, thin films, doping

## Abstract

More and more electrically conducting materials are required to sustain the technological progress of civilization. Faced with the performance limits of classical materials, the R&D community has put efforts into developing nanomaterials, which can offer sufficiently high operational parameters. In this work, single-walled carbon nanotubes (SWCNTs) were doped with polyethyleneimine (PEI) to create such material. The results show that it is most fruitful to combine these components at the synthesis stage of an SWCNT network from their dispersion. In this case, the electrical conductivity of the material is boosted from 249 ± 21 S/cm to 1301 ± 56 S/cm straightforwardly and effectively.

## 1. Introduction

The emergence of nanomaterials has revolutionized numerous fields of science and technology [1]. The key reason behind this phenomenon is that although these structures are made from well-known building blocks, once their size becomes constrained in at least one of the dimensions to 100 nm, unexpected properties are noted [2]. Depending on whether the size of a material is limited in size in one, two, or three directions, the so-called 2D, 1D, and 0D nanomaterials can be discerned, respectively [3]. This limitation makes the properties of nanomaterials very much size-dependent. For example, manipulation of the aspect ratio can drastically change their optical properties [4]. The properties of nanomaterials can be additionally tuned by changing their chemical composition.

A class of nanomaterials particularly open for tuning are nanostructures made of carbon. Organic chemistry is an excellent illustration of the capabilities of carbon, which is used in this field to construct a limitless number of chemical compounds of radically different properties. Shortly after carbon nanotubes (CNTs) [5] and graphene [6] were presented to the world at the turn of the 21st century, scientists started exploring the possibility of their modification. The main goals behind these efforts are to improve their compatibility with other materials such as polymers [7] or to modulate their electrical [8], optical [9], and mechanical properties [10], etc. Regarding the electrical characteristics, similarly to classical semiconductors, surface modification can be exercised on nanocarbon to boost its electrical conductivity via doping [11,12]. Alternatively, the doping species can also be accommodated in the inner cavity of the CNTs [13,14,15].

Many doping agents have been exploited for improving the capabilities of nanocarbon already. For instance, in the case of amphoteric CNTs, both p- and n-dopants can be employed [16]. For the former, halogens [17,18] or strong mineral acids [19,20] are usually selected. On the other hand, the latter dopant class contains electron-rich chemical compounds such as alkali metals [18,21] or nitrogen-bearing compounds [22,23]. Nitrogen-containing polymers are often engaged as they can have multiple nitrogen atoms available for doping, while they are chemically stable at the same time. It was also reported that interfacing CNTs with polyamine species can improve the mechanical properties of nanocarbon composites [24]. One of the commonly used macromolecular compounds of this sort is polyethyleneimine (PEI) [25,26,27,28,29,30]. Typically, PEI is dissolved in a suitable solvent such as water [27,28] or ethanol [26,29], and then the solution is put in contact with the CNTs so that the dopant adsorbs on their surface. Previous reports show that the electrical conductivity of CNT networks doped with PEI this way is considerably improved. However, despite the merits of this strategy, it is challenging to distribute the dopant evenly throughout the whole CNT network. For example, the dipping of CNTs in PEI solution inevitably gives the highest concentration of the dopant on the surface of the material due to mass transfer limitations for chemical compounds of such a high molecular weight. Besides that, the doping is an extra step, making the route from fabrication to the application more demanding.

In this work, composite films composed of single-walled CNTs (SWCNTs) and PEI are produced at the synthesis phase of the SWCNT ensembles to circumvent these obstacles. The PEI introduction in situ results in much higher electrical conductivity of SWCNT films than when they are doped with PEI afterward as usual. We report that applying the proposed tactic results in a notable improvement of the electrical conductivity of the SWCNT ensemble, eventually reaching 1301 ± 56 S/cm (ca. 5× the initial value). Such an enhancement is obtained after the facile adsorption of just 1 nitrogen atom from PEI per circa 330 carbon atoms during the SWCNT film formation.

## 2. Materials and Methods 

### 2.1. Chemical Compounds and Materials

SWCNTs (Tuball, OCSiAl, Luxemburg, Luxemburg), polyethyleneimine (PEI; average molecular weight of 10,000 Da; Sigma-Aldrich, St. Louis, MO, USA), acetone (Avantor, Gliwice, Poland), and toluene (Avantor, Gliwice, Poland) were all purchased. All of the chemical compounds were of p.a. class.

### 2.2. Preparation of PEI-Doped SWCNT Films

**Figure 1:**
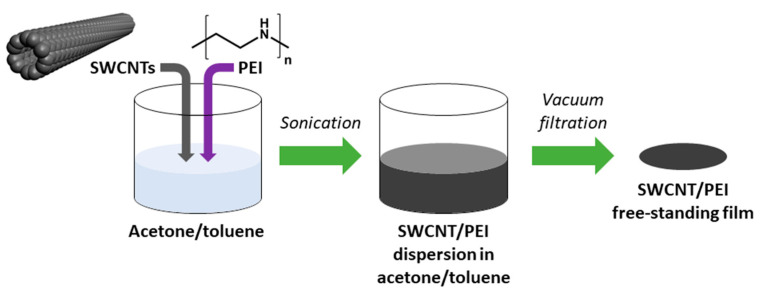
Preparation of polyethyleneimine (PEI)-doped single-walled carbon nanotube (SWCNT) free-standing films.

Film preparation methodology was an adaptation of a previously published routine for making neat SWCNT networks [31]. In brief, 145 mg of SWCNTs dried in a desiccator was introduced to 80 mL of acetone/toluene mixture (1:1, *w*/*w*). Then, 5 mg of the dopant (PEI) was added to reach the concentration of 3.33 wt. % with respect to the amount of nanocarbon. Preliminary results showed that higher ratio does not enhance the conductivity further and lower amount boosts it to a smaller extent (Figure S1). The mixture was then ultrasonicated over an ice bath with a 100% amplitude until reaching uniform dispersion (UP200St sonicator, Hielscher, Teltow, Germany). The process took 10 min. The obtained SWCNT/PEI dispersion was filtered under reduced pressure to produce a thin free-standing film of 47 mm diameter. PTFE membrane filters (pore size: 0.45 µm; Fisherbrand, Ottawa, ON, Canada) were used for this purpose. Due to the low adhesion of SWCNTs to PTFE, the material was readily delaminated from the membrane surface. Then, 3 mm × 20 mm specimens were cut out from the films for electrical characterization (Figure 1).

PEI-free SWCNT films were also produced as a reference. In that case, 145 mg of SWCNTs was subjected to the processing mentioned above as well. Then, where indicated, these ensembles were doped with a PEI solution (5 mg in 80 mL of acetone/toluene mixture, 1:1, *w*/*w*) by consecutive immersion of the film in the medium for 5 min. The specimens were dried afterward to eliminate the possible solvent effects.

### 2.3. Characterization

The microstructure of the films was studied using a scanning electron microscope (SEM, FEI Quanta 250 FEG, Hillsboro, OR, USA) at 5 kV acceleration voltage. An online detector coupled to the microscope analyzed the chemical composition of the material by means of the energy-dispersive X-ray spectroscopy (EDX) from 0 to 15 keV.

Raman spectroscopy was used to detect electronic and structural changes to the material upon doping (inVia Renishaw Raman microscope, λ = 633 nm, Wotton-under-Edge, United Kingdom) from 10 cm^−1^ to 3200 cm^−1^. I_D_/I_G_ ratios and G peak maximum positions were established and presented as mean values along with corresponding standard deviations. Twenty accumulations were recorded for each sample in multiple locations to minimize the effect of background noise and ensure the statistical significance of the collected data.

Electrical conductivity was measured using a 4-probe method with a source meter (Keithley 2450 SourceMeter, Cleveland, OH, USA). Temperature of the samples was controlled by heating the sample on a hot plate (06-MS-S280PRO, Chemland, Poland) and measuring its temperature with an infrared camera (FLIR ETS320, Wilsonville, OR, USA). Conductance was recalculated to conductivity by considering the sample area and thickness. The thickness was measured with a micrometer screw gauge (Electronic Universal IP54, Linear Tools, Cleveland, OH, USA).

## 3. Results

**Figure 2:**
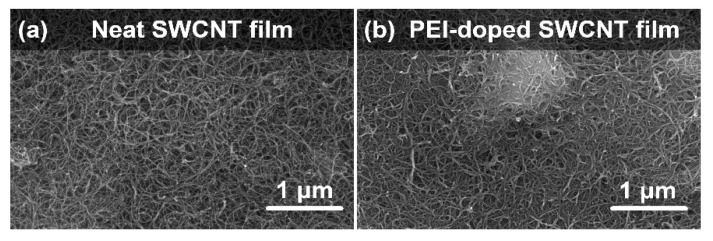
SEM micrographs of (**a**) neat and (**b**) PEI-doped SWCNT free-standing films.

At the beginning of the study, the microstructure of the SWCNT films was investigated by SEM (Figure 2). The analysis showed that the neat SWCNT film was composed of intertwined bundles of SWCNTs arranged in an isotropic fashion as expected for material prepared by unconstrained vacuum filtration. Spontaneous alignment of SWCNTs occurs only when special conditions are established, e.g., filtration rate is limited to a few mL/h of the dispersion [32,33], which was not the case herein. Regarding purity, no obvious signs of contamination were discerned in the material.

Upon doping with PEI, the microstructure of the SWCNT films stayed mostly intact. Firstly, the doping process did not introduce adulteration to the material. The absence of dopant agglomerations proves that PEI was successfully dissolved in the medium and homogeneously distributed throughout the SWCNT network. Secondly, the porous character of the material was preserved. Closer analysis of the micrographs revealed slight densification of the material. Since the size of the voids between SWCNT bundles was reduced, the material should become more conductive. A major extrinsic factor that hinders exploiting the excellent electrical conductivity of individual SWCNTs at the macroscale is the problem of the so-called junction resistance [34]. Charge propagation between SWCNTs constituting the network is challenging because they are separated by air-filled cavities, which are insulating. Therefore, the observed minute improvement to the packing degree should counteract this effect.

**Figure 3:**
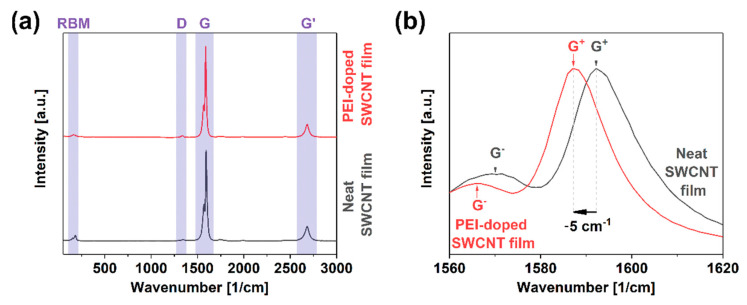
(**a**) Full Raman spectra of neat and PEI-doped SWCNT free-standing films; (**b**) close-up view on the G peak area.

Besides the extrinsic phenomenon of contact resistance, intrinsic components hamper the charge propagation through the SWCNT network. The presence of crystal defects in the form of functional groups or dislocations substantially impacts the ability of the material to transfer electrical, thermal, and mechanical energy [35]. A straightforward method to gauge the level of imperfection in nanocarbon materials is Raman spectroscopy. The ratio of intensities of defect-induced band D (sp^3^ carbon atoms) to that of the graphitic vibrations G (sp^2^ carbon atoms) quantifies this property [36]. These features result from various vibrational modes of the curved graphene lattice upon irradiation with laser light of resonance wavelength. The characterization results showed that the starting material was of very high quality as the I_D_/I_G_ ratio was as low as 0.017 ± 0.003 (Figure 3a). Furthermore, the presence of the radial breathing mode (RBM) feature corresponding to the expansion/contraction of a CNT in the radial direction confirmed that the CNTs are single-walled. After doping the SWCNT films with PEI, the ratio remained of comparable magnitude, i.e., 0.019 ± 0.004. The small discrepancy stayed within the measurement uncertainty level. Thus, it was concluded that the analyzed doping process did not deteriorate the quality of the material.

Furthermore, a detailed analysis of the G band line shape indicated that it was split into G^−^ and G^+^ components, once again providing evidence for the single-walled character of the material (Figure 3b) [37]. The shape of the G^−^ component can determine the type of SWCNTs in resonance [38,39]. In this case, the SWCNTs revealed substantial semiconducting character, which made them ideal for doping. However, taking into account the diameter distribution of the material (1.8 ± 0.4 nm [31]), metallic SWCNTs should also be excited at the selected wavelength according to the Kataura plot [40]. This fact justified why the shape of the G^−^ band was somewhat smeared on the lower wavenumber side, which could be expected for the material containing metallic SWCNTs [41].

Another vital piece of information that can be elucidated from the spectra is the position of G^+^ maximum upon doping with respect to that of the neat material. While the G+ peak position was 1592 cm^−1^ for the neat material, the feature shifted upon doping upwards by 5 cm^−1^. It was detected at 1587 cm^−1^. The shift was quite large as the dopant had to overcome the natural p-doping of the material by oxygen under ambient conditions [42]. These results give another piece of evidence that the electrical conductivity of the material should be enhanced upon the incorporation of PEI at the synthesis stage of SWCNT films.

**Figure 4:**
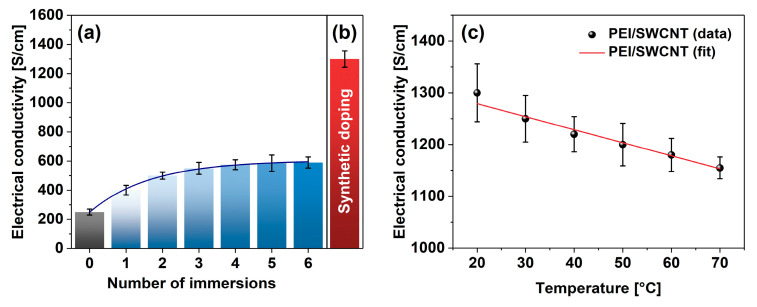
Comparison of the impact of PEI addition on electrical conductivity by (**a**) consecutive immersion and (**b**) synthetic doping. (**c**) Electrical conductivity of PEI-doped SWCNT films as a function of temperature.

To validate the reasonableness of the proposed approach, a blank experiment was carried out wherein a neat SWCNT film was consecutively doped with PEI solution for 10 min. The sample was dried afterward, its electrical conductivity was established, and then the film was subjected to the subsequent treatment. In total, the films were put through six immersion-drying-measurement cycles (Figure 4a). In the beginning, the SWCNT film had an electrical conductivity of 249 ± 21 S/cm (on par with previous results [36]), but the exposure to PEI solution gradually increased the conductivity at an exponential pace. After six doping routines, the electrical conductivity of the SWCNT films eventually reached 593 ± 21 S/cm. Therefore, the classical doping approach boosted the electrical conductivity of the SWCNT films noticeably. An enhancement by 138% was observed after such processing.

On the other hand, measurement of the electrical conductivity of the SWCNT films doped in situ at the synthesis stage revealed a clear advantage (Figure 4b). In this case, the electrical conductivity of the SWCNT-PEI films was as high as 1301 ± 56 S/cm. It amounted to the boost of the electrical conductivity by 422% in relation to the conductivity of the undoped SWCNT films determined earlier. Since the conductivity of the doped-material decreased with temperature in a reversible manner, the material exhibited predominantly metallic characteristics (Figure 4c).

To work out why doping with the same chemical compound can give radically different results, one needs to consider the doping process mechanics and its inherent constraints. The classical approach, wherein the SWCNT film is dipped in the doping solution, exhibits diffusion-related limitations. While the film is in the medium, a sizeable chemical compound such as PEI of 10,000 Da employed in the study experiences difficulties in penetrating the network. Firstly, it is bulky, so its mobility is low. Consequently, short dipping time is insufficient to enable efficient migration of these species to the inside of the SWCNT film. Secondly, once the first immersion round is conducted, the dopant can have an undesirable tendency to stack itself on the other PEI molecules present on the surface of the SWCNTs. The lack of appropriate contact between them makes the doping ineffective. 

Conversely, when PEI is injected at the beginning of the film manufacturing process, several favorable phenomena can be expected. Firstly, PEI molecules can make close contact with the surfaces of all SWCNTs individualized by sonication. Thus, the exposed de-bundled SWCNTs are evenly covered with the doping species. Even more, sonication used for dispersion of SWCNTs could facilitate penetration of the dopant species into the inner cavity to fill them [13]. Campo and colleagues reported that the material used in this study can readily accommodate water or hydrocarbons inside of it. Hence, it cannot be excluded that the employed ultrasounds facilitated migration of a part of PEI to the SWCNT interior as well. 

Therefore, the film is assembled from well-doped material. Secondly, the filtration process can liberate loosely bound redundant PEI molecules from the material, thereby improving the electrical conductivity of the network. An excessive amount of insulating dopant can prohibit effective charge transfer between individual SWCNTs in the ensemble. Therefore, it is essential not to over-dope the material, which can be avoided by the proposed approach at the selected PEI concentration. 

**Figure 5:**
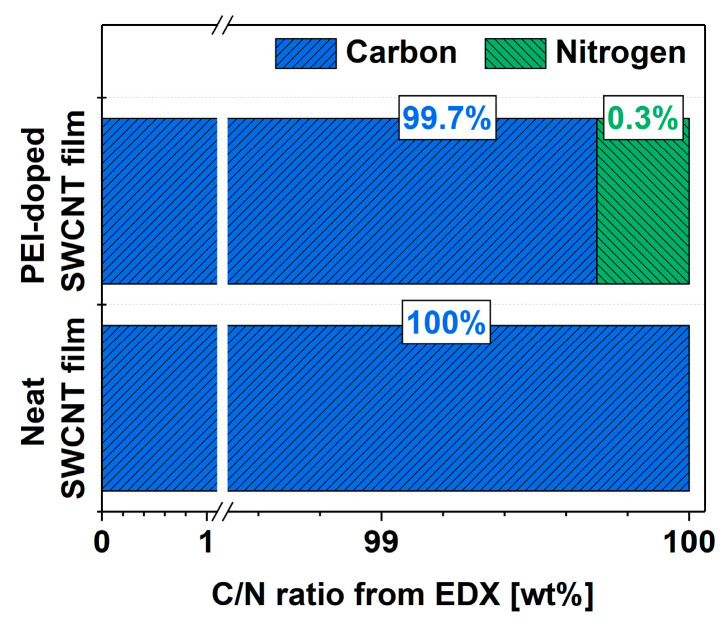
Composition of the SWCNT films before and after doping determined from EDX as C/N atomic ratio.

Finally, the goal was to determine the amount of nitrogen that caused the favorable modulation of the electrical properties of the SWCNT network. EDX was employed to determine the elemental composition of the material before and after doping with PEI (Figure 5). The results are presented as ratios of intensities of the integrated features relating to carbon and nitrogen in the spectra (at.%). In the neat SWCNT films, no nitrogen was detected, which could have originated from the synthesis, a proprietary technique undisclosed by the manufacturer. On the other hand, the doping process resulted in the detection of nitrogen in the material. The notable improvement to the electrical conductivity of the SWCNT films resulted from the incorporation of nitrogen at such a low level as 0.3% (concerning the total carbon content coming from both SWCNTs and the PEI itself). To put this in perspective, the interaction of 1 nitrogen atom from PEI per ca. 330 carbon atoms was sufficient to cause a favorable effect.

## 4. Conclusions

In summary, we demonstrated a straightforward technique of substantial enhancement of the electrical conductivity of SWCNT networks. When thin free-standing films were produced directly from SWCNT dispersion containing the PEI dopant, the electrical conductivity of the obtained material was the highest. Values as high as 1301 ± 56 S/cm were registered. Conversely, when the SWCNT films were doped in a typical dipping fashion, the electrical conductivity amounted only to 593 ± 21 S/cm.

The introduction of the doping species in situ guaranteed their uniform distribution in the whole volume of the SWCNT network. All the SWCNTs constituting the network were put in contact with the dopants, thereby boosting the ability of the material to transport charge. What is more, the addition of an appropriate amount of the dopant to the SWCNT dispersion at the beginning eliminated the possibility of over-doping of the material, which is one of the risks of the typical immersion doping methodology.

The efficacy of the proposed tactic validated the concept, simultaneously providing evidence that this method can be adapted for doping of other (nano)materials with numerous doping agents. For instance, nitrogen- and halogen-bearing compounds showed auspicious doping action of nanocarbon materials. Therefore, it would be vital to test how well these chemical species would enhance the electrical conductivity of the material when they are combined with individual SWCNTs while manufacturing macroscopic ensembles from them, not afterward.

## Data Availability

The data presented in this study are available from the corresponding author upon a reasonable request.

## Supplementary Materials

The following are available online at https://www.mdpi.com/1996-1944/14/1/65/s1, Figure S1: The impact of doping on the electrical conductivity of SWCNT films as a function of the amount of employed PEI. Films made from 145 mg of SWCNTs were immersed once in the doping solution (80 mL of acetone/toluene mixture, 1:1, *w*/*w*) containing 0.0, 0.1, 0.5, 1.0, 2.0, 5.0 and 10.0 mg of PEI.
