# Supplementary material for: Effective Doping of Single-Walled Carbon Nanotubes with Polyethyleneimine
Source: Materials (Basel). 2020 Dec 25;14(1):65. doi: 10.3390/ma14010065 (PMC7795803; doi:10.3390/ma14010065)
Supplement: Supplementary file 1 [file materials-14-00065-s001.pdf]

Supplementary Information

Effective doping of single-walled carbon nanotubes with polyethyleneimine

Monika Rdest [1], and Dawid Janas [2,*]

[1] Department of Materials Science and Metallurgy, University of Cambridge, Cambridge CB3 0FS, UK; Monika.Rdest@gmail.com
[2] Department of Organic Chemistry, Bioorganic Chemistry and Biotechnology, Silesian University of Technology, Gliwice 44-100, Poland; Dawid.Janas@polsl.pl
* Correspondence: Dawid.Janas@polsl.pl; Tel.: +48-32-237-1082 (D.J.)

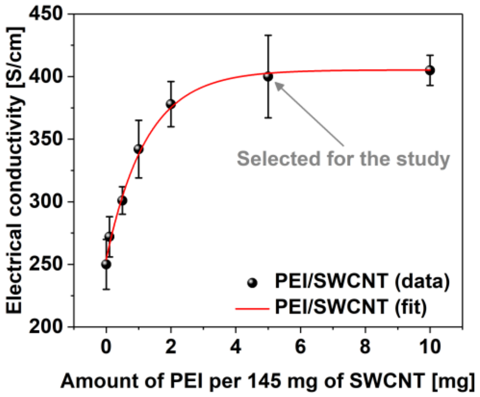

Figure S1. The impact of doping on the electrical conductivity of SWCNT films as a function of the amount of employed PEI. Films made from 145 mg of SWCNTs were immersed once in the doping solution (80 mL of acetone/toluene mixture, 1:1, w/w) containing 0.0, 0.1, 0.5, 1.0, 2.0, 5.0 and 10.0 mg of PEI.
